# Phenotypic Characterization and Genomic Mining of Uric Acid Catabolism Genes in *Lactiplantibacillus plantarum* YC

**DOI:** 10.3390/foods14244343

**Published:** 2025-12-17

**Authors:** Yuqing Zhao, Sen Yang, Miao He, Peihan Chai, Zhenou Sun, Qiaomei Zhu, Zhenjing Li, Qingbin Guo, Huanhuan Liu

**Affiliations:** 1 State Key Laboratory of Food Nutrition and Safety, College of Food Science and Engineering, Tianjin University of Science and Technology, Tianjin 300457, China; zyqrainie@163.com (Y.Z.); 15032585979@163.com (S.Y.); zhenousun@tust.edu.cn (Z.S.); qmzhu@tust.edu.cn (Q.Z.); lizhenjing@tust.edu.cn (Z.L.); guoqingbin008322@tust.edu.cn (Q.G.); 2 Key Laboratory of Digital-Intelligence and Dynamic Perception for Food Quality of China Light Industry, Beijing Technology and Business University, Beijing 100048, China; 2451031051@st.btbu.edu.cn; 3 Talent Introduction Service Center of Gongzhuling City, Gongzhuling 136100, China; chaipeihan@163.com

**Keywords:** *Lacticaseibacillus*, whole-genome sequencing, uric acid metabolism, purine metabolism, pangenome

## Abstract

This study presents the phenotypic characterization and genomic mining of uric acid catabolism genes in *Lactiplantibacillus plantarum* YC, a novel food-grade lactic acid bacterium isolated from traditional fermented vegetables with potent uric acid-lowering activity. YC is non-hemolytic, catalase- and gelatinase-negative, exhibits strong adhesion and broad antibacterial activity, and degrades 29.22% of uric acid in vitro, along with complete (100%) degradation of inosine and guanosine. Whole-genome sequencing revealed a 3,214,448 bp chromosome encoding 3026 protein-coding genes. Comparative genomics-based functional annotation highlighted abundant CAZy-related genes and antimicrobial factors, including lysozyme and monooxygenase. Crucially, genomic mining identified a complete uric acid degradation gene cluster, comprising *pucK* (uric acid permease), *hpxO* (uric acid hydroxylase), eight copies of *hiuH* (5-hydroxyisourate hydrolase), *allB* (allantoinase), and purine nucleoside transport/metabolism genes (*rihA*, *rihB*, *rihC*, *pbuG*). This work provides the first comparative genomic insight into the genetic architecture and distribution of uric acid metabolism in *L. plantarum*, elucidating YC’s dual urate-lowering mechanism and delivering key molecular markers for developing enzyme-based functional foods and microbial therapeutics against hyperuricemia.

## 1. Introduction

Hyperuricemia (HUA), a metabolic disorder resulting from dysregulated purine metabolism, is characterized by persistently elevated serum uric acid levels. With a global prevalence of 20–30% and a steadily increasing trend, HUA has become the second most common metabolic disease after diabetes [1]. It is not only the primary cause of gout but also strongly associated with numerous chronic conditions. Current management strategies are limited by drug-related adverse effects and emerging resistance, underscoring the need for safe and effective alternative interventions [2,3].

Probiotics—live microorganisms that confer health benefits to the host upon colonization—have emerged as promising candidates for managing metabolic disorders, including HUA [4]. Evidence suggests that certain probiotics can lower uric acid through multiple mechanisms: modulating gut microbiota composition, enhancing uric acid degradation, inhibiting purine synthesis, promoting renal excretion, and mitigating inflammation [5,6,7]. Among them, *Lactiplantibacillus plantarum* (*L. plantarum*), a prominent member of the lactic acid bacteria widely found in fermented foods and natural environments, exhibits exceptional probiotic properties and is frequently employed in food fermentation and functional supplements [8]. Previous studies have demonstrated that *L. plantarum* LTJ1 and LTJ48, isolated from Chinese Baijiu fermentation grains, reduce serum uric acid levels in mice by 31.0% and 51.5%, respectively [9]. While strain SQ001 was shown to regulate uric acid metabolism via its nucleoside hydrolase gene *iunH*, facilitating nucleoside uptake and hydrolysis [10].

Despite these advances, most studies on *L. plantarum*’s uric acid-lowering effects have remained at the phenotypic level, with limited genomic insight into the complete metabolic pathways and underlying molecular mechanisms. Although prior work identified *rihA*/*B*/*C*-mediated nucleoside hydrolysis through genome annotation [11], the absence of long-read sequencing data has hindered accurate assessment of genome completeness, and pan-genome analyses across strains are lacking—preventing robust evaluation of the functional specificity and evolutionary conservation of uric acid metabolism traits. This knowledge gap impedes the rational design and targeted application of next-generation probiotics for HUA management.

Whole-genome sequencing, coupled with pan-genome analysis, offers a powerful framework to dissect probiotic functionality at the molecular level. Such approaches enable systematic characterization of genomic architecture, functional gene distribution, and adaptive potential, thereby facilitating the precise identification of key enzymes and complete catabolic pathways involved in uric acid metabolism [12]. Critically, genomic mining of uric acid catabolism genes allows for the discovery of conserved or strain-specific genes responsible for direct uric acid degradation. Concurrently, comprehensive genomic screening can assess safety attributes—such as the absence of antibiotic resistance genes, virulence factors, and hemolytic activity, thereby providing a rigorous scientific basis for clinical translation [13].

In this study, we investigate *L. plantarum* YC, a strain exhibiting potent uric acid-lowering activity [14], through integrated whole-genome and pan-genome analyses. Our study not only confirms that YC harbors a complete uric acid degradation pathway that includes *pucK*, *hpxO*, eight copies of *hiuH*, *allB*, and purine nucleoside transport and metabolism genes (*rihA*, *rihB*, *rihC*, *pbuG*), but also delineates its genetic diversity and ecological adaptability within the *L. plantarum* species through comparative genomics. This work represents the first comparative genomic elucidation of the genetic basis and distribution pattern of uric acid metabolism in *L. plantarum*, offering essential molecular markers and a robust foundation for developing enzyme-driven functional foods and microbial therapeutics targeting hyperuricemia.

## 2. Materials and Methods

### 2.1. Materials

*L. plantarum* YC (CGMCC No. 25306), *Escherichia coli* ATCC 8739, *Staphylococcus aureus* ATCC 29213, *Bacillus subtilis* WB 200, and *Salmonella enteritidis* BNCC 103134 were preserved by the Laboratory of Fermented Foods and Microbial Resource Development, Tianjin University of Science and Technology. De Man, Rogosa and Sharpe (MRS) broth (BD Difco, Franklin Lakes, NJ, USA) was used for strain activation, subculturing, and seed culture preparation. A commercial biochemical identification kit for lactic acid bacteria was purchased from HaiBo Biotechnology Co., Ltd. (Qingdao, China). Columbia blood agar plates, antibiotic susceptibility disks, and Luria–Bertani (LB) agar were obtained from Biocellum Biotechnology Co., Ltd. (Changsha, China).

### 2.2. Culture and Taxonomic Identification of YC

YC was activated by two consecutive subcultures in MRS broth at 37 °C under anaerobic conditions. For experimental use, the strain was inoculated at 3% (*v*/*v*) into fresh MRS broth and incubated anaerobically at 37 °C for 20 h to prepare a standardized seed suspension. Genomic DNA was extracted, and the 16S rRNA gene was amplified using universal primers 27F and 1492R. Sanger sequencing was performed by Genewiz (Suzhou, China). The resulting 16S rDNA sequence was submitted to the NCBI BLAST server (https://blast.ncbi.nlm.nih.gov/Blast.cgi (accessed on 12 May 2022)) for homology search against the nucleotide database. A phylogenetic tree was constructed using the neighbor-joining method in MEGA 7.0 software [15], with bootstrap analysis (1000 replicates) to assess node reliability.

### 2.3. Probiotic Characterization of YC

#### 2.3.1. Physiological and Biochemical Identification

Carbohydrate fermentation profiles were determined using the lactic acid bacteria identification kit according to the manufacturer’s instructions. Catalase and gelatin liquefaction (gelatinase) tests were conducted following the protocols outlined in *Classification and Experimental Methods of Lactic Acid Bacteria* [16]. Hemolytic activity was assessed by streaking YC onto Columbia blood agar plates and incubating anaerobically at 37 °C for 48 h. *S. aureus* ATCC 29213 was included as a positive control for β-hemolysis [17].

#### 2.3.2. Auto-Aggregation and Surface Hydrophobicity Assays

Auto-aggregation capacity was evaluated according to the method of Zommiti et al. [18] with minor modifications. The YC seed culture was harvested by centrifugation (8000× *g*, 5 min), washed three times with sterile phosphate-buffered saline (PBS, pH 7.2), and resuspended to an optical density at 600 nm (OD_600_) of 0.4 (designated as *A*_0_). A 4 mL aliquot was vortexed for 10 s and incubated statically at room temperature for 3 h. The OD_600_ of the upper phase (*Aₜ*) was measured, and auto-aggregation percentage was calculated as:

(1) $$\mathrm{Auto}\text{-}\mathrm{aggregation}\;\mathrm{percentage}\;\left(\%\right)\;=\;\frac{A_{0}-A_{t}}{A_{0}}\times 100\%$$

Surface hydrophobicity was determined following Rokana et al. [19] with modifications. The bacterial suspension (OD_600_ = 0.4, *A*_0_) was mixed with chloroform (3:1, *v*/*v*), vortexed for 30 s, and allowed to stand at room temperature for 30 min to allow phase separation. The OD_600_ of the aqueous phase (*Aₓ*) was measured, and hydrophobicity was calculated as:

(2) $$\mathrm{Hydrophobicity}\;\left(\%\right)\;=\;\frac{A_{0}-A_{x}}{A_{0}}\times 100\%$$

#### 2.3.3. In Vitro Antibacterial Assay

Antibacterial activity was assessed using the agar well diffusion method [20]. Indicator pathogens—*E. coli* ATCC 8739, *S. aureus* ATCC 29213, *B. subtilis* WB 200, and *S. enteritidis* BNCC 103134—were cultured to mid-log phase (OD_600_ ≈ 1.0) and spread (100 μL) onto LB agar plates. Four 6 mm diameter wells were punched around the periphery of each plate, and 20 μL of YC suspension (1.0 × 10^9^ CFU/mL) was added to each. A central well received 20 μL of sterile 0.9% NaCl as a negative control. Plates were incubated at 37 °C for 18 h, and inhibition zone diameters (including well diameter) were measured in triplicate.

#### 2.3.4. Antibiotic Resistance Assessment

Antibiotic resistance was evaluated using the Kirby–Bauer disk diffusion method in accordance with CLSI guidelines [21]. The YC suspension (1.0 × 10^9^ CFU/mL) was spread onto MRS agar plates, and commercially available antibiotic disks (e.g., ampicillin, tetracycline, erythromycin, chloramphenicol) were placed on the inoculated surface. After incubation at 37 °C for 24 h under anaerobic conditions, the inhibition zone diameters were measured.

#### 2.3.5. In Vitro Uric Acid-Degrading Activity Assay

Uric acid-degrading activity was assessed based on the method of Cao [22] with modifications. YC cells were harvested by centrifugation, washed three times with 0.9% NaCl, and resuspended in 10 mL of 5 mM sodium urate buffer (pH 7.0). The reaction mixture was incubated anaerobically at 37 °C for 8 h. Following centrifugation (12,000× *g*, 10 min), the supernatant was filtered through a 0.22 μm membrane and analyzed by HPLC (Agilent 1260, Agilent Technologies, Inc., Santa Clara, CA, USA) using a ShimNex HE C18-AQ8 reversed-phase column (250 mm × 4.6 mm, 5 μm). The mobile phase consisted of 10 mM ammonium acetate–methanol (99:1, *v*/*v*), delivered isocratically at 1.0 mL/min. Uric acid was detected at 280 nm. A standard curve for uric acid was prepared (*Y* = 55,511*X* − 4.115, *R*^2^ = 0.9997, concentration range 0.00–0.20 mg/mL, where Y represents peak area) (Figure S1). The degradation rate was calculated as:

(3) $$\mathrm{Uric}\;\mathrm{acid}\;\mathrm{degradation}\;\mathrm{rate}\;\left(\%\right)\;=\;\frac{B_{0}-B}{B_{0}}\times 100\%$$

where *B*_0_ and *B* are the peak areas of uric acid in the supernatant in the absence and presence of the strain, respectively.

#### 2.3.6. In Vitro Purine-Degrading Activity Assay

Degradation of inosine and guanosine was evaluated following Li [23]. Washed YC cells were resuspended in 10 mL of buffer containing 1.0 mg/mL inosine and 1.0 mg/mL guanosine, and incubated anaerobically at 37 °C for 8 h. The reaction was terminated by adding 0.1 M perchloric acid (HClO_4_) at a 9:1 (*v*/*v*) ratio, followed by centrifugation and filtration (0.22 μm). HPLC analysis was performed on an Agilent 1260 system with the same C18 column, using ultrapure water–methanol (90:10, *v*/*v*) as the mobile phase at 0.8 mL/min, with detection at 254 nm, a wavelength characteristic of the purine ring system. Degradation rates were calculated as:

(4) $$\mathrm{Purine}\;\mathrm{degradation}\;\mathrm{rate}\;\left(\%\right)\;=\;\frac{C_{0}-C}{C_{0}}\times 100\%$$

where *C*_0_ and *C* represent the peak areas of inosine/guanosine in the supernatant following the reaction in the absence and presence of the strains, respectively.

### 2.4. Whole-Genome Sequencing, Assembly, and Functional Annotation of YC

High-molecular-weight genomic DNA was extracted from YC using a bacterial genomic DNA extraction kit (TIANGEN, Beijing, China) and quantified via Qubit and agarose gel electrophoresis. Whole-genome sequencing was performed by Majorbio (Shanghai, China) using a hybrid strategy: Illumina HiSeq X Ten (2 × 150 bp) for high-accuracy short reads and PacBio Sequel II for long reads to resolve repetitive regions [24]. Raw reads were quality-filtered using SeqPrep (for Illumina, San Diego, CA, USA) and SMRT Link (for PacBio, Menlo Park, CA, USA). The genome was assembled de novo using Unicycler (v0.4.8), which integrates SPAdes and Flye algorithms, yielding a complete circular chromosome and any plasmids. Assembly quality was assessed by BUSCO (using the *Lactobacillales* dataset) and read mapping back to the assembly.

Gene prediction and annotation were conducted using Prokka (v1.14.6). tRNA and rRNA genes were identified with tRNAscan-SE (v2.0) and Barrnap (v0.9), respectively. Functional annotation was performed by aligning predicted proteins against COG, Gene Ontology (GO), and KEGG databases using DIAMOND (E-value ≤ 1 × 10^−5^). Carbohydrate-active enzymes (CAZymes) were annotated via the dbCAN2 meta server (HMMER, Hotpep, and DIAMOND consensus). Antibiotic resistance genes were identified by searching against the Comprehensive Antibiotic Resistance Database (CARD, v3.0.9) using RGI (Resistance Gene Identifier) with strict detection criteria.

### 2.5. Comparative Genomic Analysis of YC

A pan-genome analysis of *L. plantarum* was conducted using a standardized pipeline. A total of 190 complete *L. plantarum* genomes (labeled “Complete” in NCBI RefSeq) were downloaded and uniformly re-annotated using Prokka (v1.14) to ensure consistency. Average nucleotide identity (ANI) was calculated using FastANI (v1.31) with a fragment length of 500 bp; strains sharing ≥ 95% ANI were considered the same species. Hierarchical clustering based on the ANI matrix was performed using Ward’s method.

The pan-genome was constructed with Roary (v3.13.0). Protein sequences from all strains were clustered using BLASTP (https://blast.ncbi.nlm.nih.gov/Blast.cgi, accessed on 12 May 2022) (E-value ≤ 1 × 10^−5^, ≥95% identity), and homologous gene families were delineated using the MCL algorithm (inflation parameter = 1.5). Core genes (present in ≥99% of strains) and accessory genes were classified accordingly. A maximum-likelihood phylogenetic tree was inferred from concatenated core genome alignments using IQ-TREE (v2.2.0) under the best-fit substitution model selected by ModelFinder. Branch support was assessed by SH-aLRT and ultrafast bootstrap (1000 replicates). Finally, pan-genome functional annotation was performed using the Prokka-CAST pipeline (40% similarity threshold), integrating evidence from 26 public databases to assign putative functions.

## 3. Results

### 3.1. Strain Identification

**Figure 1:**
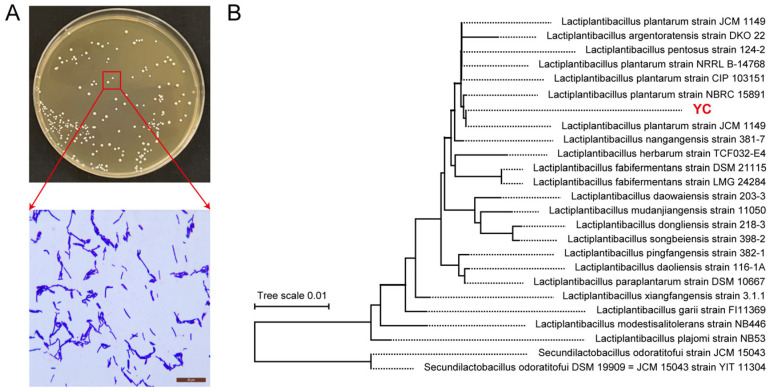
Identification of *L. plantarum* YC. (**A**) Colony morphology and Gram staining. (**B**) Phylogenetic analysis based on 16S rDNA sequences.

YC forms circular, smooth-edged colonies and stains Gram-positive (Figure 1A). Phylogenetic analysis based on 16S rRNA gene sequences using the Neighbor-Joining method in MEGA placed YC within a well-supported clade containing multiple *L. plantarum* reference strains, confirming its taxonomic assignment to the species *L. plantarum* (Figure 1B).

### 3.2. In Vitro Evaluation of Probiotic Properties

#### 3.2.1. Physiological and Biochemical Characterization

*L. plantarum* YC ferments a broad spectrum of carbohydrates, including lactose, maltose, inulin, sucrose, sorbitol, salicin, mannitol, raffinose, cellobiose, and esculin, but is unable to hydrolyze 1% sodium hippurate (Table 1). Both catalase and gelatin liquefaction tests yielded negative results, consistent with the typical biochemical profile of *L. plantarum* (e.g., strain EL2) [25,26].

**Figure 2:**
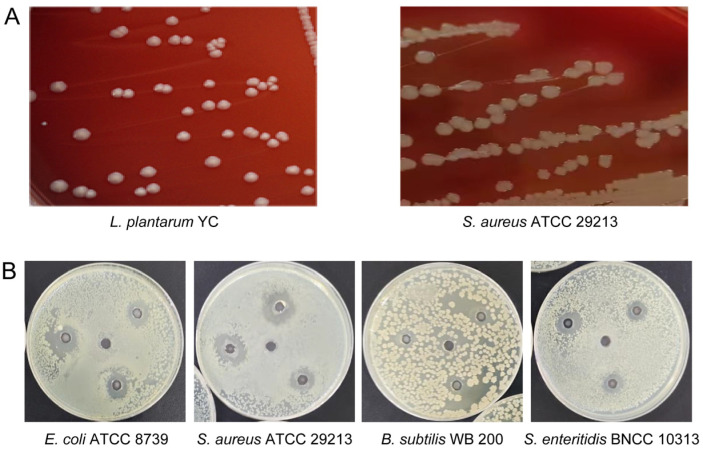
Functional characterization of *L. plantarum* YC. (**A**) Hemolytic activity assay. (**B**) Antibacterial activity against pathogenic bacteria.

Hemolysis assessment on blood agar revealed a clear β-hemolytic zone around *S*. *aureus* ATCC 29213, whereas *L. plantarum* YC exhibited no hemolytic activity (γ-hemolysis), supporting its safety for potential probiotic use [27] (Figure 2A).

#### 3.2.2. Auto-Aggregation and Surface Hydrophobicity

*L. plantarum* YC displayed high surface hydrophobicity (74.01 ± 1.23%) and an auto-aggregation rate of 50.73 ± 0.85% after 24 h, indicating strong adhesion potential and a favorable capacity for intestinal colonization.

#### 3.2.3. Antibacterial Activity

*L. plantarum* YC exhibited broad-spectrum inhibitory activity against all tested pathogens (Figure 2B). The largest inhibition zone was observed against *S. aureus* ATCC 29213 (17.88 ± 0.32 mm), followed by *E. coli* ATCC 8739 (16.18 ± 0.27 mm). A smaller, less distinct zone was seen against *Salmonella enteritidis* BNCC 103134 (12.75 ± 0.19 mm). Inhibition of *B*. *subtilis* WB 200 was characterized by an irregular, diffuse clear zone, suggesting the involvement of diffusible antimicrobial compounds.

#### 3.2.4. Antibiotic Resistance Profile

Given the potential for horizontal gene transfer of antibiotic resistance determinants from lactic acid bacteria to the human microbiota [28], antimicrobial susceptibility testing was performed. *L. plantarum* YC was susceptible to ampicillin, erythromycin, rifampicin, lincomycin, and chloramphenicol; resistant to streptomycin, kanamycin, norfloxacin, ciprofloxacin, tetracycline, and vancomycin; and showed intermediate susceptibility to imipenem (Table 2). This profile aligns with intrinsic resistance patterns commonly observed in lactobacilli [29].

#### 3.2.5. In Vitro Uric Acid-Reducing Ability

Following 8 h of incubation, *L. plantarum* YC degraded 29.22% of exogenous uric acid (Figure S2). Notably, it completely degraded both inosine and guanosine within 1 h, achieving 100.00% degradation for each purine nucleoside (Table 3, Figure S3). This dual activity—direct uric acid catabolism coupled with efficient precursor clearance—suggests the presence of a coordinated enzymatic system for purine metabolism, which was subsequently confirmed through genomic mining.

### 3.3. Genomic Features and Functional Annotation

**Figure 3:**
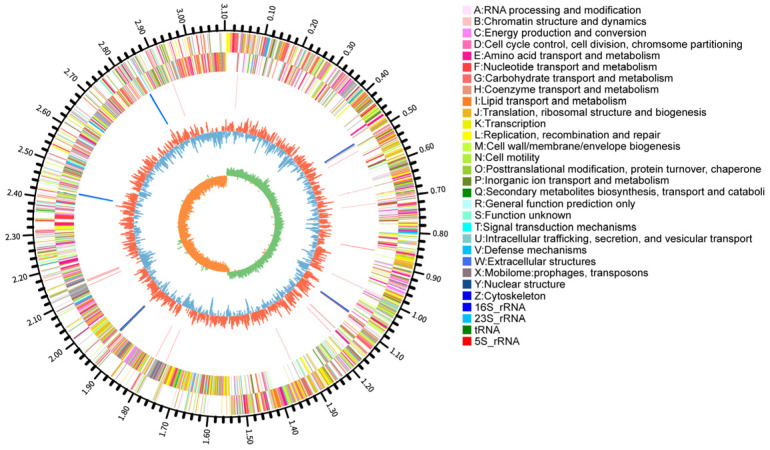
Circular genomic map of *L. plantarum* YC.

The complete genome of *L. plantarum* YC comprises a single circular chromosome (3,214,448 bp; GC content: 44.52%) and five plasmids (58,438, 38,011, 8700, 3209, and 2008 bp) (Figure 3). It harbors 3026 predicted genes, including 2943 protein-coding sequences (CDS), 67 tRNAs, and 16 rRNAs. The average gene length is 890.64 bp, with a gene density of 0.94 genes/kb and a coding density of 83.84%. Additionally, 72 repetitive elements were identified (48 tandem and 24 interspersed repeats).

### 3.4. Functional Gene Annotation

**Figure 4:**
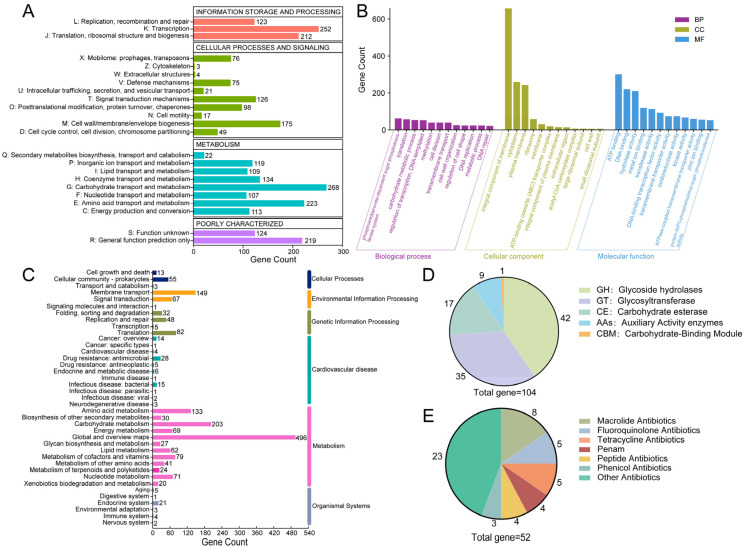
Functional annotation of the *L. plantarum* YC genome. (**A**) COG categories. (**B**) GO terms. (**C**) KEGG pathways. (**D**) CAZy family annotation. (**E**) Classification of antibiotic resistance genes.

Of the 2943 CDS, 2398, 2278, and 1476 were annotated in the COG, GO, and KEGG databases, respectively. COG classification revealed enrichment in carbohydrate transport and metabolism (268 genes), transcription (218), and amino acid transport and metabolism (195) (Figure 4A). Critically, 107 genes were assigned to nucleotide transport and metabolism, providing a genomic basis for *L. plantarum* YC’s efficient degradation of purine nucleosides like inosine and guanosine.

GO analysis highlighted enrichment in biological processes such as the phosphoenolpyruvate-dependent sugar phosphotransferase system, translation, and carbohydrate metabolism; cellular components were predominantly membrane- and ribosome-associated; molecular functions were dominated by catalytic, transporter, and binding activities (Figure 4B).

KEGG pathway mapping confirmed *L. plantarum* YC’s robust capacity for core metabolic functions, including carbohydrate and amino acid metabolism, membrane transport, and protein synthesis (Figure 4C), all essential for survival in the gut environment.

### 3.5. Specialized Metabolic Features

#### 3.5.1. CAZyme-Encoding Genes

*L*. *plantarum* genomes are enriched in carbohydrate-active enzyme (CAZyme) genes, which encode a diverse array of enzymes capable of systematically degrading complex polysaccharides and dietary fibers. This genomic feature enables the bacterium to efficiently utilize dietary fibers present in the gut environment, thereby supporting its colonization, metabolic activity, and probiotic functionality within the intestinal ecosystem [30].

*L*. *plantarum* YC encodes 104 carbohydrate-active enzymes (CAZymes), with glycoside hydrolases (GHs) being the most abundant (42 genes, 40.38% of total CAZymes) (Figure 4D). Notably, GH25 and GH73 families include genes encoding lysozyme (EC 3.2.1.17) [31], which hydrolyzes peptidoglycan and contributes to *L. plantarum* YC’s antibacterial activity—consistent with the inhibition zones observed in Figure 2B.

Additionally, *L*. *plantarum* YC harbors genes for auxiliary activity (AA) enzymes: *laccase* (AA1; EC 1.10.3.2) involved in polyphenol oxidation, and copper-dependent lytic polysaccharide monooxygenases (LPMOs) from AA10 (e.g., chitin monooxygenase EC 1.14.99.53 and cellulose monooxygenase EC 1.14.99.54). This copper-containing polyphenol oxidoreductase is involved in the degradation of polymers such as lignin and humic substances [32]. These enzymes enhance dietary fiber utilization and may contribute to antifungal effects, reinforcing *L. plantarum* YC’s probiotic functionality [33,34].

#### 3.5.2. Antibiotic Resistance Genes

A total of 52 antibiotic resistance genes were identified (Figure 4E), primarily conferring resistance to macrolides (8 genes) and fluoroquinolones (5 genes). Although certain antimicrobial resistance determinants were identified in the genome, phenotypic susceptibility testing confirmed that *L. plantarum* YC remains susceptible to β-lactams, macrolides, rifamycins, chloramphenicol, and penems. Given the inherent differences between genotypic and phenotypic assessment methods, the presence of resistance determinants does not necessarily imply phenotypic resistance or susceptibility in the isolate [35]. Moreover, strain YC may exhibit susceptibility to these antibiotics due to gene silencing or genetic loss, which is a common feature among commensal lactobacilli [36].

### 3.6. Comparative Genomics

#### 3.6.1. Dataset and Core Genome Metrics

**Figure 5:**
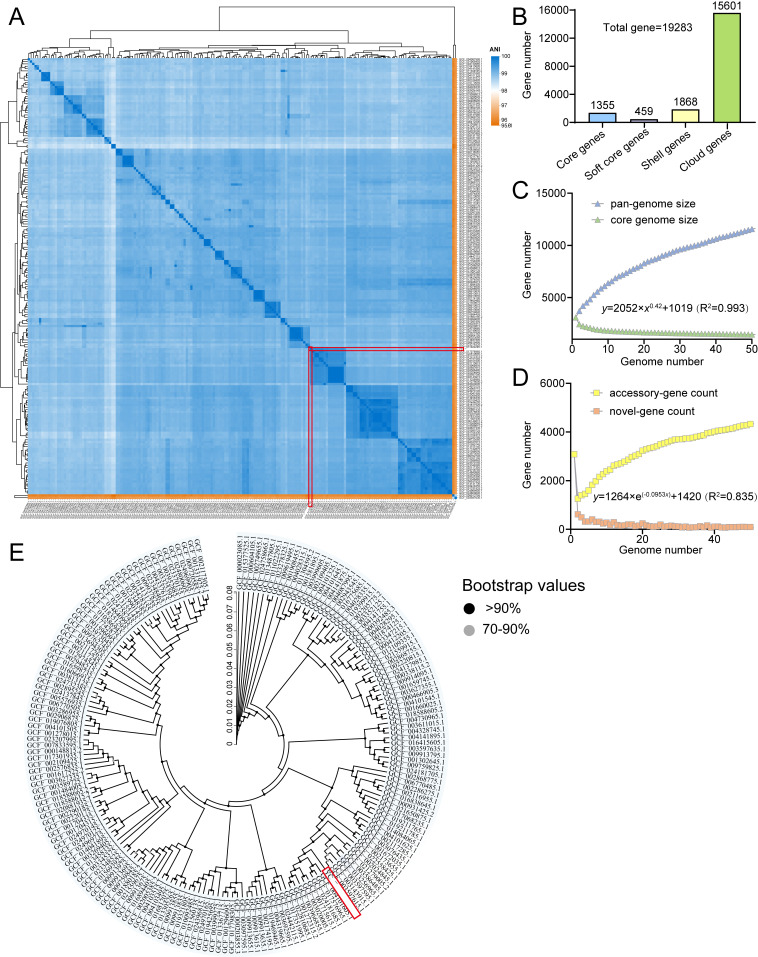
Comparative genomic analysis of *L. plantarum*. (**A**) ANI-based heatmap. (**B**) Pan-genome statistics. (**C**,**D**) Pan/core and unique/new gene accumulation curves. (**E**) Core genome phylogeny. Target strain YC is highlighted with a red box.

Pan-genome analysis was performed on 190 complete *L. plantarum* genomes, including *L. plantarum* YC (Table S1). Genome sizes ranged from 2.952 to 3.697 Mb (mean: 3.314 Mb), with an average of 3144 genes per strain. ANI analysis confirmed high genomic similarity among all strains, with YC clustering tightly within the *L. plantarum* species (Figure 5A).

#### 3.6.2. Pan-Genome Dynamics

The pan-genome is open, comprising 19,283 non-redundant genes, of which 1355 (7.03%) constitute the core genome (Figure 5B). The pan-genome curve shows no sign of saturation, and the number of strain-specific (unique) genes continues to increase with each added genome (Figure 5C,D), reflecting extensive genomic plasticity and adaptive potential through horizontal gene transfer [37].

#### 3.6.3. Core Genome-Based Phylogeny

A high-confidence phylogenetic tree (bootstrap > 90%) based on the core genome confirmed *L. plantarum* YC’s close evolutionary relationship with other *L. plantarum* strains and validated the robustness of the pan-genome dataset (Figure 5E).

### 3.7. Genomic Mining of Uric Acid Catabolism Genes

**Figure 6:**
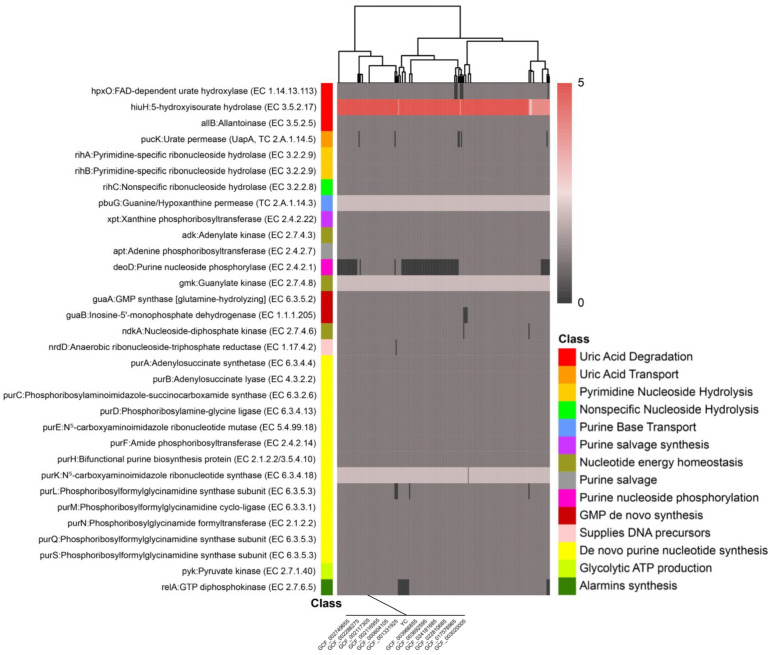
Distribution and conservation of key uric acid metabolism-related genes across 190 *L. plantarum* genomes.

To systematically dissect the molecular basis of uric acid metabolism in *L*. *plantarum*, we performed a comprehensive genomic mining of purine metabolic genes across a population of 190 strains using presence/absence variation (PAV) analysis of the core and accessory genomes. This approach revealed a conserved genetic architecture underlying uric acid catabolism, with strain YC harboring a functionally coherent pathway (Figure 6; Table S2).

Specifically, the *L*. *plantarum* YC genome contains the gene *hpxO* encoding FAD-dependent uric acid hydroxylase (Uox) at locus LAHLIFBI_02707, eight copies of the gene *hiuH* encoding 5-hydroxyisourate hydrolase (HiuH), and the gene *allB* encoding allantoinase (AllB) at locus LAHLIFBI_02273 (Table S2). The *hpxO* gene product converts uric acid to 5-hydroxyisourate in the presence of the cofactor flavin adenine dinucleotide (FAD). This intermediate is then hydrolyzed to allantoin by HiuH, which is subsequently converted to allantoic acid by AllB. Finally, allantoic acid is degraded to CO_2_ and NH_3_ through sequential catalysis by allantoicase and urease, completing the uric acid degradation pathway [38]. The enzymes encoded by the *hpxO*, *hiuH*, and *allB* genes enable *L. plantarum* YC to form a complete uric acid metabolic pathway, providing new potential for microbial modulation directly targeting uric acid.

Comparative analysis across the *L. plantarum* population revealed that the *hpxO*, *hiuH*, and *allB* gene loci are also present in some other strains. This indicates metabolic pathway conservation between *L. plantarum* YC and its related strains, suggesting that the species may exhibit similar expression of uric acid-lowering genes or a specific bias toward the purine metabolism pathway. This conservation suggests a potential species-level predisposition toward purine catabolism in certain lineages, possibly linked to ecological niche adaptation.

In parallel, *L. plantarum* YC also modulates uric acid levels through precursor clearance and metabolic redirection. The genome encodes key components of the purine salvage pathway, including purine nucleoside phosphorylase (*deoD*), xanthine phosphoribosyltransferase (*xpt*), and adenine phosphoribosyltransferase (*apt*). These enzymes facilitate the recycling of free purine bases (e.g., hypoxanthine, xanthine, adenine) into nucleotides, thereby diverting purine flux away from the oxidative degradation pathway that culminates in uric acid production. Notably, 80 of the 190 analyzed genomes lack *deoD*, which disrupts the salvage pathway and likely shunts purine nucleosides toward uric acid generation [39]. In contrast, *L. plantarum* YC’s intact *deoD* supports efficient nucleoside recycling, contributing to its low uric acid output phenotype.

Furthermore, *L. plantarum* YC is equipped with specialized transport and hydrolysis systems for extracellular purine nucleosides. It carries the uric acid permease gene *pucK*, encoding a uric acid/H^+^ symporter that mediates the cellular uptake of uric acid and xanthine [40]. Additionally, *L. plantarum* YC harbors three ribonucleoside hydrolase genes: *rihA* and *rihB* (pyrimidine-preferring but with broad specificity) and *rihC* (nonspecific), which collectively hydrolyze extracellular inosine and guanosine into their respective purine bases. These bases are then imported via the guanine/hypoxanthine permease PbuG, encoded by *pbuG* [11]. This coordinated system explains *L. plantarum* YC’s observed 100% degradation of inosine and guanosine within 1 h (Table 3).

In addition to the aforementioned genes, the *L. plantarum* YC genome encodes key enzymes of the purine salvage pathway, including xanthine phosphoribosyltransferase (*xpt*) and adenine phosphoribosyltransferase (*apt*), which enable recycling of free purine bases and directly reduce the conversion of purines into uric acid. Additionally, genes such as *guaB*, encoding inosine-5′-monophosphate dehydrogenase, and *purB*, encoding adenylosuccinate lyase, regulate nucleotide turnover, thereby inhibiting de novo purine biosynthesis. Genes such as *guaA* (encoding GMP synthase), *ndkA* (nucleoside diphosphate kinase), and *purA* (adenylosuccinate synthetase) accelerate nucleotide recycling, indirectly reducing the accumulation of uric acid precursors (IMP, XMP).

Collectively, these genomic features reveal that *L. plantarum* YC employs a dual, synergistic strategy to lower uric acid: (1) direct enzymatic degradation of uric acid via a complete catabolic pathway, and (2) clearance of purine nucleoside precursors coupled with redirection of purine flux through the salvage pathway rather than the oxidative route. This integrated metabolic architecture provides a robust molecular foundation for *L. plantarum* YC’s uric acid–lowering phenotype and positions it as a promising candidate for the development of next-generation probiotics targeting hyperuricemia and related metabolic disorders.

## 4. Discussion

In this study, we performed a comprehensive phenotypic characterization and whole-genome analysis of *L. plantarum* YC. Beyond confirming its favorable probiotic traits, our high-resolution genomic mining revealed a complete and functionally coherent uric acid degradation pathway—the first such pathway identified to date in a food-grade lactic acid bacterium. *L. plantarum* YC demonstrates a direct enzymatic capacity to catabolize uric acid and its precursors, offering a concrete molecular basis for its urate-lowering activity and highlighting its potential as a rationally designed functional microbe for metabolic health applications [41].

The safety profile of *L. plantarum* YC aligns well with established standards for probiotic use. It exhibited no hemolytic activity and tested negative for catalase and gelatinase production. Furthermore, it remained susceptible to several clinically relevant antibiotics, including ampicillin, erythromycin, rifampicin, and chloramphenicol—consistent with the generally recognized safety of *L. plantarum* strains [42]. Functionally, YC displayed high surface hydrophobicity (74.01 ± 1.23%) and auto-aggregation capacity (50.73 ± 0.85%), traits that support robust adhesion to intestinal surfaces and potential colonization. It exhibited broad-spectrum antibacterial activity against common foodborne pathogens, including *S. aureus*, *E. coli*, and *S. enteritidis*. When applied as a bioprotective culture in fermented foods, *L. plantarum* YC enhances food safety, extends shelf life, and reduces contamination risk through its ability to inhibit pathogenic bacteria. Furthermore, within the gut ecosystem, this antagonistic activity helps maintain microbial community balance by suppressing the overgrowth of harmful bacteria, thereby promoting overall gastrointestinal health. This attribute underscores the considerable potential of *L. plantarum* YC as a next-generation probiotic, demonstrating significant integrated benefits for both food preservation and host health.

HPLC analysis confirmed that *L. plantarum* YC degraded uric acid by 29.22% in vitro and completely metabolized purine nucleosides. While excluding an internal standard limits absolute precision, the methodology provides a robust basis for the reported results. The high linearity (*R*^2^ = 0.9997) of the validated external standard curve ensures reliable relative quantification for uric acid. Meanwhile, the 100% degradation of inosine and guanosine is confirmed by the unequivocal endpoint of complete substrate depletion (peak disappearance). This qualitative assessment is considered sufficient to demonstrate metabolic capacity, bypassing the need for complex quantitative calculations.

Genomic annotation provides a mechanistic explanation: YC is enriched in CAZymes, notably lysozyme from the GH25 family and copper-dependent lytic polysaccharide monooxygenases (LPMOs) from the AA10 family. These enzymes not only facilitate the utilization of complex dietary fibers, thereby supporting gut persistence, but also contribute to antibacterial effects through pepti-doglycan hydrolysis, particularly against Gram-positive bacteria.

While several *L. plantarum* strains with uric acid-modulating potential have been reported—such as X7022 (via purine assimilation) [43]—a host-derived strain acting through nucleoside hydrolases [10] and K-Mar-A2 exhibiting uricase-like activity [44] none have been shown to possess a complete uric acid catabolic pathway. For instance, *L. plantarum* MC14 encodes multiple nucleoside hydrolases and transporters but lacks downstream genes required for uric acid degradation [45]. In contrast, *L. plantarum* YC harbors a fully assembled gene cluster for uric acid catabolism, including *pucK* (uric acid permease), *hpxO* (FAD-dependent uric acid hydroxylase), eight copies of *hiuH* (5-hydroxyisourate hydrolase), and *allB* (allantoinase). This enzymatic cascade converts uric acid sequentially into 5-hydroxyisourate, allantoin, and allantoic acid, ultimately yielding CO_2_ and NH_3_ [46].

Concurrently, *L. plantarum* YC encodes a dedicated system for purine nucleoside processing: the permease *pbuG* and three ribonucleoside hydrolases (*rihA*, *rihB*, *rihC*) enable rapid uptake and hydrolysis of extracellular inosine and guanosine, consistent with the observed 100% degradation within one hour. Critically, *L. plantarum* YC also maintains a functional purine salvage pathway, featuring key genes such as *deoD*, *xpt*, *apt*, *guaA*, *guaB*, and *purA*. This network efficiently recycles free purine bases into nucleotides, thereby minimizing flux through the oxidative branch of purine metabolism that leads to uric acid production. The coexistence of direct uric acid catabolism and precursor diversion via salvage constitutes a dual, synergistic mechanism that enhances the strain’s overall urate-lowering efficacy.

Pan-genome analysis based on 190 *L. plantarum* strains systematically elucidated the species-wide context and evolutionary significance of *L. plantarum* YC’s uric acid metabolism. Core genes *hpxO*, *hiuH*, and *allB* are notably conserved in some strains, suggesting a potential purine metabolism bias or adaptation to purine-rich ecological niches within the species. In contrast, *L. plantarum* YC possesses both a complete direct degradation pathway and an efficient precursor-processing module, resulting in a synergistic enhancement of its uric acid-lowering function. The open pan-genome structure of *L. plantarum* confers persistent potential for horizontal gene transfer, explaining its metabolic diversity and providing ample engineering potential for the rational design and enhancement of *L. plantarum* YC’s uric acid metabolism pathway through synthetic biology approaches.

Although the complete uric acid degradation gene cluster was clearly annotated based on sequence homology and synteny analysis, this study did not include direct experimental validation of gene expression or enzyme activity. While the significant in vitro uric acid-degrading capacity observed in the *L. plantarum* YC strain strongly suggests the functionality of this metabolic pathway, future studies should focus on verifying the transcriptional activation and catalytic activities of these key enzymes to definitively establish their role in the observed phenotype.

## 5. Conclusions

This study confirms through in vitro evaluation that *L. plantarum* YC possesses excellent probiotic properties, exhibiting significant uric acid-degrading activity and complete degradation of guanosine and inosine. Whole-genome analysis reveals that *L. plantarum* YC has robust carbohydrate metabolism capabilities and possesses genes associated with antibacterial activity, while its open pan-genome structure contributes to strong environmental adaptability. *L. plantarum* YC possesses a complete molecular mechanism for uric acid degradation. The genes *pucK*, *hpxO*, *hiuH*, and *allB* act synergistically to form a complete metabolic pathway from uric acid to allantoin, providing the genetic basis for the strain’s direct uric acid degradation capability. With the deep integration of food enzyme engineering and synthetic biology, naturally efficient catalytic strains like *L. plantarum* YC are poised to become core components of next-generation precision nutrition foods, driving the evolution of functional foods.

## 6. Patents

Li, Z.J.; Chai, P.H.; Liu, H.H.; Guo, Q.P.; Wang, X.Y. *Lactiplantibacillus plantarum* strain with uric acid-degrading activity, probiotic composition comprising same and uses thereof. (In Chinese) CN Patent Application No. CN115786187A, Publication Date: 14 March 2023.

## Figures and Tables

**Table 1:** Physiological and Biochemical Characteristics.

Test Item		YC *
Carbohydrate Type	Lactose	+
	Maltose	+
	Inulin	+
	Sucrose	+
	Sorbitol	+
	Salicin	+
	Mannitol	+
	Raffinose	+
	Cellobiose	+
	Esculin	+
	1% Sodium Hippurate	−
Additional Reactions	Catalase Reaction	−
	Gelatin Liquefaction	−

* “+” indicates a positive result; “–” indicates a negative result.

**Table 2:** Assessment of Antibiotic Resistance.

Type	Antibiotics	Disk Potency (μg)	Susceptibility *
β-Lactam	Ampicillin	10.00	S
Aminoglycoside	Streptomycin	10.00	R
Aminoglycoside	Kanamycin	30.00	R
Macrolide	Erythromycin	10.00	S
Fluoroquinolone	Norfloxacin	10.00	R
Fluoroquinolone	Ciprofloxacin	5.00	R
Rifamycin	Rifampicin	5.00	S
Lincosamide	Lincomycin	2.00	S
Chloramphenicol	Chloramphenicol	30.00	S
Carbapenem	Imipenem	10.00	I
Tetracycline	Tetracycline	30.00	R
Polypeptide	Vancomycin	30.00	R

* “S” indicates susceptible; “I” indicates intermediate susceptibility; “R” indicates resistant.

**Table 3:** Assimilation of Uric Acid and Nucleosides by Strain YC.

Type	Uric Acid	Inosine	Guanosine
Degradation Rate (%)	29.22	100.00	100.00

## Data Availability

The original contributions presented in this study are included in the article/Supplementary Materials. Further inquiries can be directed to the corresponding author.

## Supplementary Materials

The following supporting information can be downloaded at: https://www.mdpi.com/article/10.3390/foods14244343/s1, Table S1: Summary Table of Genomic Information of Strain; Table S2: Summary Table of Key Genes in Microbial Uric Acid Metabolism. Figure S1. HPLC chromatograms of uric acid at different concentrations: (A) 0.06 mg/mL, (B) 0.09 mg/mL, (C) 0.15 mg/mL, and (D) 0.20 mg/mL. Figure S2. HPLC chromatograms showing uric acid degradation by *Lactiplantibacillus plantarum* YC. (A) Chromatogram of the reaction mixture before incubation with strain YC. (B) Chromatogram after incubation with strain YC. Figure S3. HPLC chromatograms showing the degradation of purine nucleosides by *Lactiplantibacillus plantarum* YC. (A) Chromatogram of the reaction mixture before incubation with strain YC. (B) Chromatogram after incubation with strain YC.

## Abbreviations

The following abbreviations are used in this manuscript:

HUA	Hyperuricemia
COG	Clusters of Orthologous Groups of proteins
GO	Gene Ontology
KEGG	Kyoto Encyclopedia of Genes and Genomes
CAZyme	Carbohydrate-Active enZYME
*L. plantarum*	*Lactiplantibacillus plantarum*
*E. coli*	*Escherichia coli*
*B. subtilis*	*Bacillus subtilis*
*S. enteritidis*	*Salmonella enteritidis*
*S. aureus*	*Staphylococcus aureus*
