# Supplementary material for: Phenotypic Characterization and Genomic Mining of Uric Acid Catabolism Genes in Lactiplantibacillus plantarum YC
Source: Foods. 2025 Dec 17;14(24):4343. doi: 10.3390/foods14244343 (PMC12732358; doi:10.3390/foods14244343)
Supplement: Supplementary file 1 [file foods-14-04343-s001.zip › foods-3981920-supplementary.pdf]

Table S1. Summary Table of Genomic Information of Strain

Taxon	Strain Name	Taxonomy ID	Assembly No.	GenBank Accession No.	Contig N50 (Mb)	Genome Size (Mb)	Gene Number	Submission Date
Lactiplantibacillus plantarum	ZJ316	1284663	ASM33811v2	GCF_000338115.2	3.204	3.300	3,136	2013/7/31
	WCFS1	220668	ASM20385v3	GCF_000203855.3	3.308	3.349	3,159	2011/8/1
	P-8	767468	ASM39248v2	GCF_000392485.3	3.036	3.247	3,112	2015/1/23
	CGMCC1.557	337330	ASM127231v2	GCF_001272315.2	3.157	3.273	3,106	2017/7/5
	TS12	337330	ASM190845v1	GCF_001908455.1	3.193	3.434	3,293	2016/12/19
	SRCM100434	337330	ASM217419v1	GCF_002174195.1	3.224	3.224	2,994	2017/6/6
	LB1-2	337330	ASM290687v1	GCF_002906875.1	3.359	3.542	3,413	2018/1/30
	nF1-FD	337330	ASM395288v1	GCF_003952885.1	3.122	3.196	2,999	2018/12/18
	SKO-001	337330	ASM557693v1	GCF_005576935.1	3.199	3.381	3,230	2019/5/22
	G1	337330	ASM1325696v1	GCF_013256965.1	3.192	3.230	3,035	2020/6/3
	M19	337330	ASM1858860v2	GCF_018588605.2	3.153	3.511	3,388	2022/4/4
	M17	337330	ASM1858861v2	GCF_018588615.2	3.245	3.294	3,098	2022/4/4
	M8	337330	ASM1858866v2	GCF_018588665.2	3.244	3.294	3,092	2022/4/4
	GR1184	337330	ASM1921176v1	GCF_019211765.1	3.209	3.306	3,120	2021/7/14
	GR1187	337330	ASM1921178v1	GCF_019211785.1	3.209	3.332	3,149	2021/7/14

GR1186	337330	ASM2165087v1	GCF_021650875.1	3.209	3.306	3,103	2022/1/27
ST-III	889932	ASM14881v1	GCF_000148815.2	3.254	3.308	3,120	2011/7/29
JDM1	644042	ASM2308v1	GCF_000023085.1	3.198	3.198	3,023	2009/7/17
DOMLa	1300221	ASM60410v1	GCF_000604105.1	3.199	3.210	3,023	2014/3/28
2025	1385856	ASM46690v3	GCF_000466905.3	3.189	3.466	3,347	2021/6/28
16	1327988	ASM41220v1	GCF_000412205.1	3.045	3.361	3,190	2013/6/18
B21	1590	ASM93142v2	GCF_000931425.2	3.284	3.311	3,111	2018/2/6
5-2	1590	ASM127801v1	GCF_001278015.1	3.238	3.238	3,044	2015/9/2
ZS2058	1590	ASM129609v1	GCF_001296095.1	3.198	3.198	2,980	2015/9/22
HFC8	1590	ASM130264v1	GCF_001302645.1	3.068	3.406	3,345	2015/9/29
WLPL04	1590	ASM133192v2	GCF_001331925.2	3.142	3.142	2,958	2018/10/18
LZ95	1590	ASM148400v1	GCF_001484005.1	3.261	3.322	3,149	2016/1/4
Zhang-LL	1590	ASM158189v1	GCF_001581895.1	2.952	2.952	2,763	2016/3/8
JBE245	1590	ASM159609v1	GCF_001596095.1	3.263	3.263	3,068	2016/3/22
CAUH2	1590	ASM161752v2	GCF_001617525.2	3.255	3.275	3,083	2018/7/10
LZ206	1590	ASM165974v1	GCF_001659745.1	3.213	3.264	3,159	2016/6/8
LZ227	1590	ASM166002v1	GCF_001660025.1	3.132	3.425	3,339	2016/6/8
NCU116	1590	ASM167203v1	GCF_001672035.1	3.355	3.355	3,192	2016/6/20
KP	1590	ASM170431v1	GCF_001704315.1	3.418	3.693	3,605	2016/8/12

DF	1590	ASM170433v1	GCF_001704335.1	3.424	3.697	3,617	2016/8/12
LY-78	1590	ASM171561v1	GCF_001715615.1	3.119	3.129	2,965	2016/8/29
C410L1	1590	ASM187412v1	GCF_001874125.1	3.098	3.393	3,245	2016/11/10
MF1298	1590	ASM188018v2	GCF_001880185.2	3.236	3.576	3,407	2018/8/17
RI-113	1590	ASM199014v1	GCF_001990145.1	3.249	3.463	3,344	2017/2/7
10CH	1590	ASM200538v2	GCF_002005385.2	3.311	3.311	3,090	2017/10/10
CLP0611	1590	ASM202484v1	GCF_002024845.1	3.198	3.231	3,039	2017/3/10
JBE490	1590	ASM210940v1	GCF_002109405.1	3.189	3.197	3,039	2017/4/25
LP2	1590	ASM210942v1	GCF_002109425.1	3.285	3.285	3,100	2017/4/25
BLS41	1590	ASM211695v1	GCF_002116955.1	3.249	3.476	3,304	2017/5/2
TMW1.25	1590	ASM211724v1	GCF_002117245.1	3.145	3.352	3,207	2017/5/2
TMW1.277	1590	ASM211726v1	GCF_002117265.1	3.099	3.400	3,240	2017/5/2
TMW1.708	1590	ASM211728v1	GCF_002117285.1	3.133	3.246	3,052	2017/5/2
TMW1.1623	1590	ASM211730v1	GCF_002117305.1	3.142	3.333	3,152	2017/5/2
SRCM102022	1590	ASM217365v1	GCF_002173655.1	3.252	3.331	3,177	2017/6/6
LPL-1	1590	ASM220577v2	GCF_002205775.2	3.187	3.201	3,029	2017/6/27
dm	1590	ASM222017v1	GCF_002220175.1	3.326	3.326	3,158	2017/7/17
GB-LP1	1590	ASM222081v1	GCF_002220815.1	3.040	3.040	2,863	2017/7/18
LP3	1590	ASM228627v1	GCF_002286275.1	3.260	3.330	3,145	2017/9/5

BDGP2	1590	ASM229018v1	GCF_002290185.1	3.407	3.582	3,468	2017/9/12
PC520	1590	ASM257683v1	GCF_002576835.1	3.343	3.453	3,293	2017/10/18
ATCC8014	1590	ASM274965v1	GCF_002749655.1	3.203	3.309	3,140	2017/11/3
KC3	1590	ASM286875v1	GCF_002868755.1	3.175	3.330	3,174	2018/1/9
K259	1590	ASM286877v1	GCF_002868775.1	3.312	3.373	3,182	2018/1/9
LM1004	1590	ASM289524v1	GCF_002895245.1	3.199	3.199	3,028	2018/1/22
X7021	1590	ASM294354v1	GCF_002943545.1	3.191	3.407	3,239	2017/12/23
KC28	1590	ASM294821v1	GCF_002948215.1	3.292	3.292	3,136	2018/2/19
K25	1590	ASM302000v1	GCF_003020005.1	3.176	3.412	3,243	2018/3/27
plantarum	1590	ASM307643v1	GCF_003076435.1	3.164	3.164	2,965	2018/5/1
LQ80	1590	ASM309759v1	GCF_003097595.1	3.230	3.448	3,276	2018/5/8
HAC01	1590	ASM314391v1	GCF_003143915.1	3.198	3.231	3,037	2018/5/20
SK151	1590	ASM326940v1	GCF_003269405.1	3.231	3.231	3,017	2018/6/28
DSR_M2	1590	ASM328695v1	GCF_003286955.1	3.183	3.251	3,056	2018/7/3
nF1	1590	ASM332539v1	GCF_003325395.1	3.121	3.192	2,997	2018/7/17
TMW1.1478	1590	ASM334537v1	GCF_003345375.1	3.326	3.387	3,212	2018/7/30
b-2	1590	ASM335212v1	GCF_003352125.1	3.211	3.211	3,041	2018/8/1
IDCC3501	1590	ASM342835v1	GCF_003428355.1	3.243	3.243	3,052	2018/8/26
DR7	1590	ASM358648v1	GCF_003586485.1	3.198	3.231	3,036	2018/9/21

ZFM55	1590	ASM358972v1	GCF_003589725.1	3.269	3.412	3,235	2018/9/23
ATG-K6	1590	ASM359759v1	GCF_003597595.1	3.206	3.263	3,074	2018/9/26
ATG-K8	1590	ASM359761v1	GCF_003597615.1	3.221	3.276	3,085	2018/9/26
ATG-K2	1590	ASM359763v1	GCF_003597635.1	3.035	3.175	3,012	2018/9/26
NCIMB700965	1590	ASM361101v1	GCF_003611015.1	3.015	3.216	3,122	2018/10/3
ZFM9	1590	ASM362733v1	GCF_003627335.1	3.262	3.434	3,262	2018/10/8
ZFM4	1590	ASM362735v1	GCF_003627355.1	3.213	3.304	3,149	2018/10/8
KACC92189	1590	ASM369259v1	GCF_003692595.1	3.241	3.460	3,256	2018/10/29
LMT1-48	1590	ASM381312v1	GCF_003813125.1	3.168	3.281	3,153	2018/11/21
SN35N	1590	ASM396685v1	GCF_003966855.1	3.268	3.415	3,314	2018/8/10
FBL-3a	1590	ASM399927v1	GCF_003999275.1	3.232	3.232	3,038	2019/1/8
Q7	1590	ASM399960v1	GCF_003999605.1	2.952	2.982	2,800	2019/1/8
YW11	1590	ASM402829v1	GCF_004028295.1	2.992	3.249	3,102	2019/1/17
13_3	1590	ASM402831v1	GCF_004028315.1	2.992	3.274	3,104	2019/1/17
12_3	1590	ASM402833v1	GCF_004028335.1	3.213	3.404	3,277	2019/1/17
SRCM103295	1590	ASM408799v1	GCF_004087995.1	3.106	3.448	3,288	2019/1/21
SRCM103311	1590	ASM410132v1	GCF_004101325.1	3.192	3.441	3,268	2019/1/22
SRCM103357	1590	ASM410150v1	GCF_004101505.1	3.277	3.543	3,343	2019/1/22
SRCM103361	1590	ASM410154v1	GCF_004101545.1	3.164	3.277	3,132	2019/1/22

SRCM103362	1590	ASM410160v1	GCF_004101605.1	3.039	3.118	3,007	2019/1/22
SRCM103418	1590	ASM410162v1	GCF_004101625.1	3.116	3.321	3,157	2019/1/22
SRCM103426	1590	ASM410164v1	GCF_004101645.1	3.183	3.481	3,299	2019/1/22
SRCM103472	1590	ASM410349v1	GCF_004103495.1	3.302	3.302	3,079	2019/1/23
SRCM103473	1590	ASM410351v1	GCF_004103515.1	3.302	3.317	3,106	2019/1/23
SRCM103297	1590	ASM414175v1	GCF_004141755.1	3.099	3.329	3,165	2019/2/6
SRCM103300	1590	ASM414187v1	GCF_004141875.1	3.179	3.388	3,240	2019/2/6
SRCM103303	1590	ASM414189v1	GCF_004141895.1	3.108	3.301	3,160	2019/2/6
IRG1	1590	ASM431966v1	GCF_004319665.1	3.224	3.379	3,167	2019/2/28
NCIMB700965.EF.A	1590	ASM432874v1	GCF_004328745.1	3.015	3.217	3,123	2019/3/6
EM	1590	ASM433761v1	GCF_004337615.1	3.185	3.649	3,484	2019/3/11
UNQLp11	1590	ASM473096v1	GCF_004730965.1	3.535	3.535	3,402	2019/4/8
pc-26	1590	ASM677048v1	GCF_006770485.1	3.182	3.303	3,095	2019/7/9
LLY-606	1590	ASM677050v1	GCF_006770505.1	3.194	3.259	3,068	2019/7/9
Y44	1590	ASM783359v1	GCF_007833595.1	3.256	3.307	3,099	2019/8/5
TMW1.1308	1590	ASM961949v1	GCF_009619495.1	3.222	3.334	3,147	2019/11/7
KCCP11226	1590	ASM972058v1	GCF_009720585.1	3.206	3.382	3,232	2019/11/30
83-18	1590	ASM975982v1	GCF_009759825.1	3.081	3.364	3,244	2019/12/17
123-17	1590	ASM975984v1	GCF_009759845.1	3.191	3.239	3,017	2019/12/17

8P-A3	1590	ASM976274v1	GCF_009762745.1	3.323	3.333	3,140	2019/12/18
SRCM100438	1590	ASM991361v1	GCF_009913615.1	3.224	3.224	2,992	2020/1/22
SRCM100440	1590	ASM991363v1	GCF_009913635.1	3.224	3.224	2,992	2020/1/22
SRCM100442	1590	ASM991365v1	GCF_009913655.1	3.224	3.224	3,002	2020/1/22
SRCM100995	1590	ASM991367v1	GCF_009913675.1	3.193	3.478	3,264	2020/1/22
SRCM101105	1590	ASM991369v1	GCF_009913695.1	3.198	3.344	3,118	2020/1/22
SRCM101187	1590	ASM991379v1	GCF_009913795.1	2.997	3.097	2,917	2020/1/22
SRCM101222	1590	ASM991383v1	GCF_009913835.1	3.171	3.358	3,172	2020/1/22
SRCM101518	1590	ASM991385v1	GCF_009913855.1	3.213	3.416	3,248	2020/1/22
SRCM102737	1590	ASM991397v1	GCF_009913975.1	3.250	3.378	3,183	2020/1/22
SRCM101167	1590	ASM991409v1	GCF_009914095.1	3.226	3.411	3,248	2020/1/22
SRCM101511	1590	ASM993782v1	GCF_009937825.1	3.074	3.273	3,064	2020/1/28
CACC558	1590	ASM1009248v1	GCF_010092485.1	3.250	3.349	3,149	2020/2/1
202195	1590	ASM1058694v1	GCF_010586945.1	3.296	3.356	3,164	2020/2/14
X7022	1590	ASM1102229v1	GCF_011022295.1	3.085	3.244	3,055	2020/2/26
LS/07	1590	ASM1130459v2	GCF_011304595.2	3.182	3.428	3,270	2022/5/9
SPC-SNU72-2	1590	ASM1210935v1	GCF_012109355.1	3.037	3.254	3,096	2020/4/7
AMT74419	1590	ASM1297454v1	GCF_012974545.1	3.227	3.227	3,029	2020/5/5
CNEI-KCA4	1590	ASM1315514v1	GCF_013155145.1	3.328	3.328	3,176	2020/5/24

TCI507	1590	ASM1330526v1	GCF_013305265.1	3.172	3.265	3,049	2020/6/9
Heal19	1590	ASM1336771v1	GCF_013367715.1	3.266	3.367	3,158	2020/6/23
HC-2	1590	ASM1345833v1	GCF_013458335.1	3.244	3.365	3,186	2020/7/22
BK-021	1590	ASM1348780v1	GCF_013487805.1	3.352	3.470	3,330	2020/7/23
CNEI-KCA5	1590	ASM1369430v1	GCF_013694305.1	3.206	3.206	3,050	2020/7/26
SK156	1590	ASM1404189v1	GCF_014041895.1	3.231	3.231	3,011	2020/8/2
BCC9546	1590	ASM1408406v1	GCF_014084065.1	3.219	3.324	3,136	2020/8/6
DSM20174	1590	ASM1413173v1	GCF_014131735.1	3.243	3.250	3,060	2020/8/10
PMO08	1590	ASM1484099v1	GCF_014840995.1	3.248	3.318	3,153	2020/10/4
TK-P2A	1590	ASM1537752v1	GCF_015377525.1	3.200	3.211	3,033	2020/11/12
ZDY2013	1590	ASM1569392v1	GCF_015693925.1	3.304	3.304	3,100	2020/11/29
PC518	1590	ASM1606691v1	GCF_016066915.1	3.143	3.434	3,290	2020/12/15
SHY21-2	1590	ASM1641560v1	GCF_016415605.1	3.099	3.334	3,183	2020/12/28
PC518	1590	ASM1659873v1	GCF_016598735.1	3.143	3.451	3,308	2021/1/10
S58	1590	ASM1677568v1	GCF_016775685.1	3.152	3.269	3,164	2021/2/1
CXG9	1590	ASM1681207v1	GCF_016812075.1	3.292	3.411	3,241	2021/2/7
KM2	1590	ASM1683864v1	GCF_016838645.1	3.233	3.418	3,243	2021/2/9
Lp900	1590	ASM1689440v1	GCF_016894405.1	3.207	3.366	3,171	2021/2/16
GR0128	1590	ASM1706823v1	GCF_017068235.1	3.211	3.333	3,147	2021/2/28

12	1590	ASM1730193v1	GCF_017301935.1	3.257	3.311	3,126	2021/3/8
AR195	1590	ASM1735199v1	GCF_017351995.1	3.219	3.364	3,172	2021/3/14
KLDS1.0386	1590	ASM1757696v1	GCF_017576965.1	3.025	3.274	3,136	2021/3/24
LRCC5314	1590	ASM1774287v1	GCF_017742875.1	3.250	3.250	3,076	2021/4/4
NCIMB8826	1590	ASM1779830v1	GCF_017798305.1	3.308	3.344	3,158	2021/4/6
ATCC202195	1590	ASM1835129v1	GCF_018351295.1	3.295	3.354	3,174	2021/5/13
XJ25	1590	ASM1907680v1	GCF_019076805.1	3.170	3.210	3,003	2021/6/30
L75a	1590	ASM1932180v1	GCF_019321805.1	3.151	3.360	3,248	2021/7/22
41P	1590	ASM1939991v1	GCF_019399915.1	3.211	3.271	3,147	2021/7/29
DW12	1590	ASM1942569v1	GCF_019425695.1	3.218	3.218	3,026	2021/7/30
MSD1	1590	ASM1946946v1	GCF_019469465.1	3.080	3.080	2,916	2021/8/9
ZW5	1590	ASM2088193v1	GCF_020881935.1	3.293	3.428	3,253	2021/11/16
022AE	1590	ASM2127900v2	GCF_021279005.2	3.234	3.234	3,050	2022/3/16
P9	1590	ASM2155967v1	GCF_021559675.1	3.185	3.388	3,182	2022/1/24
A8	1590	ASM2155991v1	GCF_021559915.1	3.038	3.219	3,063	2022/1/24
W2	1590	ASM2156013v1	GCF_021560135.1	3.255	3.255	3,062	2022/1/24
ST	1590	ASM2255842v1	GCF_022558425.1	3.059	3.321	3,159	2022/3/14
SCB0151	1590	ASM2281068v1	GCF_022810685.1	3.157	3.214	3,069	2022/4/4
LP-F1	1590	ASM2320799v1	GCF_023207995.1	3.258	3.311	3,133	2022/5/2

MNCW_1	1590	ASM2327832v1	GCF_023278325.1	3.036	3.287	3,150	2022/5/9
VHProbiV38	1590	ASM2334721v1	GCF_023347215.1	3.041	3.198	3,050	2022/5/12
LPC904	1590	ASM2334838v1	GCF_023348385.1	3.275	3.275	3,103	2022/5/12
LPIMC513	1590	ASM2334846v1	GCF_023348465.1	3.206	3.206	3,050	2022/5/12
LPT52	1590	ASM2334852v1	GCF_023348525.1	3.273	3.273	3,066	2022/5/12
JB-1	1590	ASM2337015v1	GCF_023370155.1	3.208	3.315	3,117	2022/5/15
Z.6-1	1590	ASM2397304v1	GCF_023973045.1	3.324	3.333	3,148	2022/6/28
DM083	1590	ASM2413784v1	GCF_024137845.1	3.197	3.197	3,036	2022/7/5
3-1	1590	ASM2413798v1	GCF_024137985.1	3.210	3.451	3,364	2022/7/5
P9	1590	ASM2418168v1	GCF_024181685.1	3.017	3.314	3,183	2022/7/7
Lp-6	1590	ASM2418170v1	GCF_024181705.1	3.101	3.483	3,409	2022/7/7
HOM3204	1590	ASM2439681v1	GCF_024396815.1	3.233	3.298	3,124	2022/7/25
LpYC41	1590	ASM2444211v1	GCF_024442115.1	3.249	3.303	3,126	2022/7/26
Q180	1590	ASM2473238v1	GCF_024732385.1	3.197	3.197	3,057	2022/8/22
VHProbiO04	1590	ASM2475866v1	GCF_024758665.1	3.230	3.303	3,138	2022/8/29
VHProbiO10	1590	ASM2475874v1	GCF_024758745.1	3.248	3.267	3,095	2022/8/29
SRCM210459	1590	ASM2480060v1	GCF_024800605.1	3.263	3.263	3,103	2022/8/31
SRCM210576	1590	ASM2496971v1	GCF_024969715.1	3.256	3.256	3,096	2022/9/6
SRCM210465	1590	ASM2496990v1	GCF_024969905.1	3.264	3.264	3,104	2022/9/6

	SRCM210579	1590	ASM2497012v1	GCF_024970125.1	3.256	3.256	3,103	2022/9/6
	SRCM210580	1590	ASM2497014v1	GCF_024970145.1	3.256	3.256	3,104	2022/9/6
	SRCM210797	1590	ASM2497016v1	GCF_024970165.1	3.226	3.226	3,019	2022/9/6

Table S2. Summary Table of Key Genes in Microbial Uric Acid Metabolism

Enzyme	Gene	GenBank Accession No.	Strain Name	Gene Locus
		GCF_001581895.1	Zhang_LL	AAKCHHKE_02605
		GCF_016415605.1	SHY21_2	ADLELPIA_02732
		GCF_001908455.1	TS12	AFHLPKCN_01740
		GCF_004028335.1	12_3	AKAOIJGO_00821
		GCF_009913855.1	SRCM101518	AKOKFEBJ_02423
		GCF_004101325.1	SRCM103311	ALHBBJMI_00317
		GCF_012109355.1	SPC_SNU72_2	ALKJOLKM_01362
		GCF_009913635.1	SRCM100440	AMGMDNEH_02120
FAD-dependent urate hydroxylase	*hpxO*	GCF_009913675.1	SRCM100995	AMINKCLD_00935
		GCF_021560135.1	W2	ANKMOKPI_02777
		GCF_001296095.1	ZS2058	AOJMHFAI_02003
		GCF_017742875.1	LRCC5314	BAFINJIH_01623
		GCF_009914095.1	SRCM101167	BANBIOPA_01486
		GCF_001617525.2	CAUH2	BBOKDCCD_02777
		GCF_001659745.1	LZ206	BCBFHOGE_02836
		GCF_024758745.1	VHProbiO10	BDGJKCLA_02764
		GCF_004101605.1	SRCM103362	BDOCICOH_02740

GCF_019399915.1	41P	BGOHEINB_02789
GCF_013487805.1	BK_021	BMDPOFOE_01692
GCF_015377525.1	TK_P2A	BMJMLGHN_02713
GCF_003020005.1	K25	BMKOILDP_02805
GCF_002117285.1	TMW1.708	BNFBIHII_02712
GCF_003589725.1	ZFM55	BNLBELID_00496
GCF_014131735.1	DSM20174	BNMKGLNK_00346
GCF_003611015.1	NCIMB700965	BNNIAPKF_01347
GCF_023348385.1	LPC904	CADHLLED_02800
GCF_001596095.1	JBE245	CBJBIFED_02789
GCF_004319665.1	IRG1	CBPIEIAN_02719
GCF_020881935.1	ZW5	CCNICGLJ_02823
GCF_003428355.1	IDCC3501	CGAHMEBF_02302
GCF_003627355.1	ZFM4	CGCHIDAD_01718
GCF_001880185.2	MF1298	CHKBNDNK_02735
GCF_000203855.3	NCIMB8826.WCFS1	CKFDGIGD_02798
GCF_002943545.1	X7021	CMPOMNPO_02767
GCF_006770485.1	pc_26	CNDGDIHB_02698
GCF_014041895.1	SK156	DAMKNEDF_01605

	GCF_009913975.1	SRCM102737	DBJHNPLP_02269
	GCF_013694305.1	CNEI_KCA5	DCJPBJOB_02821
	GCF_001484005.1	LZ95	DCNEKHGM_00497
	GCF_002220815.1	GB_LP1	DEHJACEK_02674
	GCF_019469465.1	MSD1	DFDGLKID_02600
	GCF_014840995.1	PMO08	DFJIHPAI_01900
	GCF_001272315.2	CGMCC1.557	DGGIMEGN_02018
	GCF_004101545.1	SRCM103361	DJFINPGP_00297
	GCF_002286275.1	LP3	DMEGDIHP_02783
	GCF_017301935.1	12	DNLPJFAK_01425
	GCF_002290185.1	BDGP2	DOCCLFGL_02278
	GCF_009913835.1	SRCM101222	DPDDGPDL_01376
	GCF_001990145.1	RI_113	EADNBLLN_03036
	GCF_024800605.1	SRCM210459	EAHILGDG_00347
	GCF_009937825.1	SRCM101511	EBPGKDKP_01289
	GCF_023278325.1	MNCW_1	EGMCBLBG_00322
	GCF_000604105.1	DOMLa	EIADAHNN_02714
	GCF_000392485.3	plantarum	EIGPLPAE_02656
	GCF_002005385.2	10CH	ELMFFKGO_02800

	GCF_001278015.1	5_2	EMPAKPMD_02792
	GCF_024137985.1	3_1	ENGONNAG_02913
	GCF_013367715.1	Heal19	EOGOOHGM_02792
	GCF_002749655.1	ATCC8014	EPDGBLBG_02714
	GCF_001672035.1	NCU116	FCKIJNHN_01469
	GCF_016812075.1	CXG9	FCLFKOOG_02842
	GCF_024969715.1	SRCM210576	FHBLBPHE_00327
	GCF_024970125.1	SRCM210579	FIAFBHII_02797
	GCF_000412205.1	16	FIJBKOLI_02629
	GCF_004141875.1	SRCM103300	FJKPEEPN_00315
	GCF_013305265.1	TCI507	FKKJOLLM_00688
	GCF_003586485.1	DR7	FMCONMPN_01766
	GCF_012974545.1	AMT74419	GBFAHMJP_02761
	GCF_002117305.1	TMW1.1623	GBGCANHH_02722
	GCF_004028315.1	13_3	GEIGDEFG_00292
	GCF_016894405.1	Lp900	GEPBBAEO_02711
	GCF_017576965.1	KLDS1.0386	GFHHCACC_02639
	GCF_021650875.1	GR1186	GFJPBKNO_02705
	GCF_001704315.1	KP	GHPOCCKE_01044

	GCF_002116955.1	BLS41	GJCNBBDF_01593
	GCF_021559675.1	P9	GJJNPDIC_02670
	GCF_017798305.1	NCIMB8826	GLABJCDA_02796
	GCF_018351295.1	ATCC202195	GLJBNIJB_02822
	GCF_023370155.1	JB_1	GLOBMBLF_02725
	GCF_013458335.1	HC_2	GODNMKIP_01564
	GCF_024969905.1	SRCM210465	GPODOIEO_00345
	GCF_003597615.1	ATG_K8	HAJJGFMM_02521
	GCF_015693925.1	ZDY2013	HBNLKFEE_02807
	GCF_003076435.1	P-8	HDOKKABM_02799
	GCF_009619495.1	TMW1.1308	HFDDJHAG_02771
	GCF_001874125.1	C410L1	HGIPIGFK_02731
	GCF_000148815.2	ST_III	HJKGJCKJ_02769
	GCF_006770505.1	LLY_606	HMAJCFNK_02748
	GCF_000338115.2	ZJ316	HMKGHILC_03048
	GCF_003952885.1	nF1_FD	HNAPHEBJ_02732
	GCF_002220175.1	dm	HNOBAMEC_02568
	GCF_018588615.2	M17	IDELAEJE_02758
	GCF_021279005.2	022AE	IDHFJKMI_01144

GCF_002174195.1	SRCM100434	IDMPOFAK_00326
GCF_002906875.1	LB1_2	IFENEKMH_02633
GCF_013155145.1	CNEI_KCA4	IGEPENGK_02999
GCF_004328745.1	NCIMB700965.EF.A	IGKBGPDE_02334
GCF_023207995.1	LP_F1	IGOKDKNO_01591
GCF_003692595.1	KACC92189	IIPKKNOM_01882
GCF_024181685.1	P9	IJLNMFOG_02188
GCF_023348465.1	LPIMC513	IMMFFKBG_02805
GCF_002109425.1	LP2	INCFDJCB_02817
GCF_010586945.1	202195	IONFGCNF_02870
GCF_003269405.1	SK151	IPKPBJOK_02404
GCF_022810685.1	SCB0151	IPPNADDA_02771
GCF_009759845.1	123_17	JAKHDGDO_02673
GCF_014084065.1	BCC9546	JBANMJBO_02773
GCF_024970165.1	SRCM210797	JBIFFBGN_02719
GCF_003999605.1	Q7	JBLLIKKD_00264
GCF_016598735.1	PC518	JEALJFBL_02717
GCF_005576935.1	SKO_001	JEHLJEFC_02822
GCF_023347215.1	VHProbiV38	JGGDPDAN_02644

	GCF_004101505.1	SRCM103357	JOJCOEJG_02791
	GCF_004103515.1	SRCM103473	JPCMBIDM_02808
	GCF_002576835.1	PC520	JPHLKKOC_03236
	GCF_004087995.1	SRCM103295	KAMJMNHD_02715
	GCF_004103495.1	SRCM103472	KCBDMBPO_00336
	GCF_018588665.2	M8	KCKLOJHK_02752
	GCF_004028295.1	YW11	KECGNCIO_00294
	GCF_002109405.1	JBE490	KFDNOKPJ_02833
	GCF_003286955.1	DSR_M2	KHEHPCKE_02766
	GCF_003966855.1	SN35N	KIDFFGKE_01928
	GCF_003999275.1	FBL_3a	KIJMKOFH_02845
	GCF_016838645.1	KM2	KLLLMDHH_02775
	GCF_007833595.1	Y44	KMEJEDJA_02769
	GCF_009720585.1	KCCP11226	KMMGGNEK_02793
	GCF_011304595.2	LS_07	KNODAOFP_02827
	GCF_003597595.1	ATG_K6	KPNJMDIG_00039
	GCF_002948215.1	KC28	LBINIBLH_02311
	GCF_003627335.1	ZFM9	LCEPCJON_02848
	GCF_021559915.1	A8	LCFIHKOG_02659

	GCF_002868775.1	K259	LHAACFNG_02548
	GCF_004141755.1	SRCM103297	LJLLIBGN_00300
	GCF_004337615.1	EM	LLDKCIHD_02784
	GCF_018588605.2	M19	LPJMKONA_02788
	GCF_009762745.1	8P_A3	MADOJEAC_02855
	GCF_003352125.1	b_2	MBIICEKP_02794
	GCF_024137845.1	DM083	MCLEJDMO_02798
	GCF_016066915.1	PC518	MEJPIEMB_02716
	GCF_009913795.1	SRCM101187	MEKHADKI_00766
	GCF_019321805.1	L75a	MGBGCCEN_02046
	GCF_003143915.1	HAC01	MKOPFDGM_01974
	GCF_000023085.1	JDM1	MNKPDIIC_02713
	GCF_004101625.1	SRCM103418	MNMIGELB_00374
	GCF_004730965.1	UNQLp11	MOFEFGJK_00423
	GCF_016775685.1	S58	MOIMBDEC_02807
	GCF_002895245.1	LM1004	MONLCDIJ_01679
	GCF_002117265.1	TMW1.277	MOPCBEPI_02707
	GCF_009913655.1	SRCM100442	MPHGGEFC_00936
	GCF_003345375.1	TMW1.1478	NACPNIEI_02884

	GCF_011022295.1	X7022	NBIICGCM_02674
	GCF_017068235.1	GR0128	NCGEEMNE_01668
	GCF_013256965.1	G1	NFJOKMHJ_00042
	GCF_002024845.1	CLP0611	NHFMKKOM_02781
	GCF_001704335.1	DF	NHIMHFGK_01425
	GCF_004101645.1	SRCM103426	NJECPOIC_02825
	GCF_009913695.1	SRCM101105	NKMKDLBO_01576
	GCF_024758665.1	VHProbiO04	NLMOKMGG_02747
	GCF_017351995.1	AR195	NNKGDJAM_02720
	GCF_001331925.2	WLPL04	NOLJINHL_02714
	GCF_024970145.1	SRCM210580	NPCDAEHJ_02797
	GCF_024732385.1	Q180	NPLBNPGN_00502
	GCF_024181705.1	Lp_6	OCCBHFKO_02776
	GCF_001715615.1	LY_78	OEFLCKON_02175
	GCF_004141895.1	SRCM103303	OEPCPPLA_00299
	GCF_000931425.2	B21	OKDOODGO_02793
	GCF_003325395.1	nF1	OLHALDNH_00053
	GCF_024396815.1	HOM3204	OMBBOECM_02768
	GCF_022558425.1	ST	OOHCPJJE_02604

		GCF_002205775.2	LPL_1	OPFFPKKG_00262
		GCF_023973045.1	Z.6_1	OPLPOBLD_02854
		GCF_001660025.1	LZ227	PCCLPAPL_02730
		GCF_019076805.1	XJ25	PCEGINEI_02672
		GCF_003097595.1	LQ80	PDEGAPHI_02755
		GCF_023348525.1	LPT52	PEAPPJGO_02780
		GCF_019211765.1	GR1184	PEHEKOEG_02705
		GCF_019211785.1	GR1187	PGGAPLFN_02707
		GCF_010092485.1	CACC558	PGJMGKCO_02377
		GCF_000466905.3	2025	PGMPMCNB_02857
		GCF_002117245.1	TMW1.25	PHPKLFKP_02744
		GCF_001302645.1	HFC8	PJNLFGAF_01562
		GCF_002173655.1	SRCM102022	PKDGMGKG_02788
		GCF_019425695.1	DW12	PMGHDLLH_02748
		GCF_009759825.1	83_18	PMOCHCCG_02697
		GCF_002868755.1	KC3	PNDPFPIM_01459
		GCF_024442115.1	LpYC41	PNMBCNFJ_02774
5-hydroxyisourate hydrolase	*hiuH*	GCF_019469465.1	MSD1	DFDGLKID_00734
		GCF_004337615.1	EM	LLDKCIHD_03139

	GCF_003597635.1	ATG_K2	PMFMELII_03032
	GCF_009759825.1	83_18	PMOCHCCG_03241

A

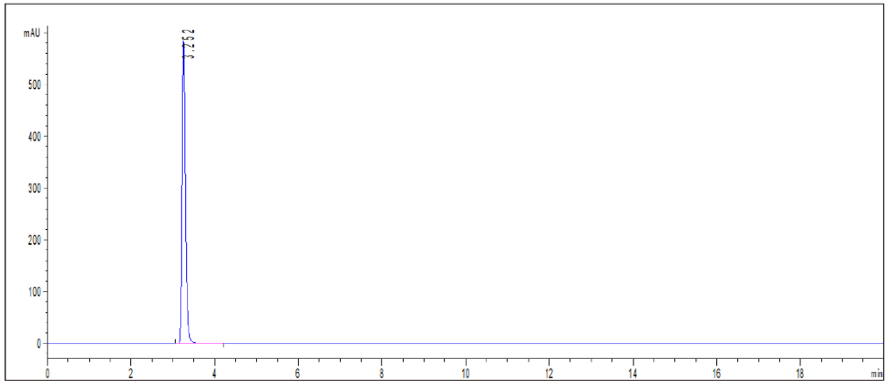

Retention time	Peak area	Peak height	Peak width	Symmetry factor	Relative peak area (%)
3.252	3234.2	583.9	0.0835	0.681	100.000

B

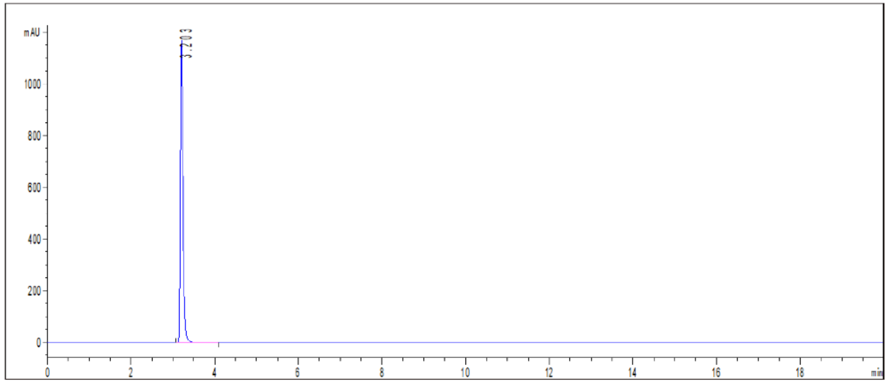

Retention time	Peak area	Peak height	Peak width	Symmetry factor	Relative peak area (%)
3.203	5105.2	1168.7	0.0655	0.636	100.000

C

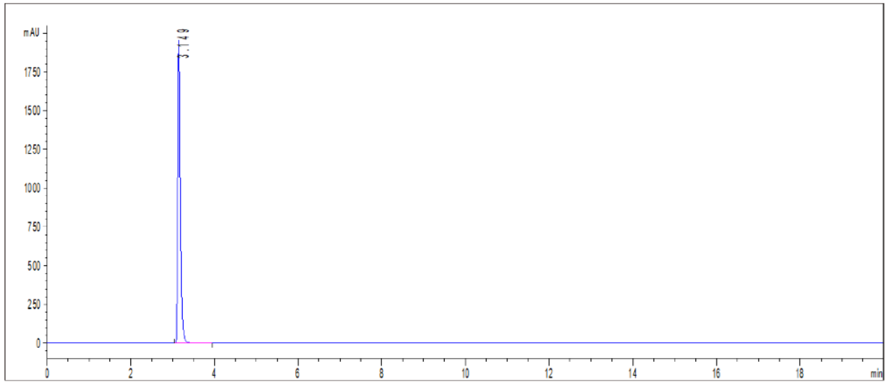

Retention time	Peak area	Peak height	Peak width	Symmetry factor	Relative peak area (%)
3.149	8315.3	1954.4	0.0642	0.655	100.000

D

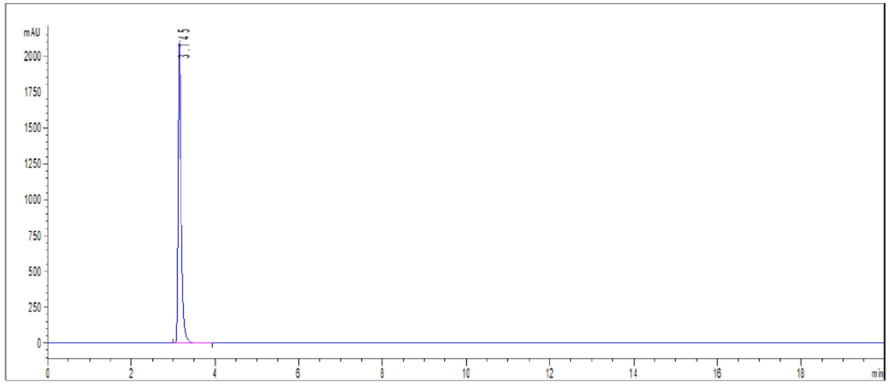

Retention time	Peak area	Peak height	Peak width	Symmetry factor	Relative peak area (%)
3.145	11080.2	2108.9	0.0788	0.624	100.000

Figure S1. HPLC chromatograms of uric acid at different concentrations: (A) 0.06 mg/mL, (B) 0.09 mg/mL, (C) 0.15 mg/mL, and (D) 0.20 mg/mL.

A

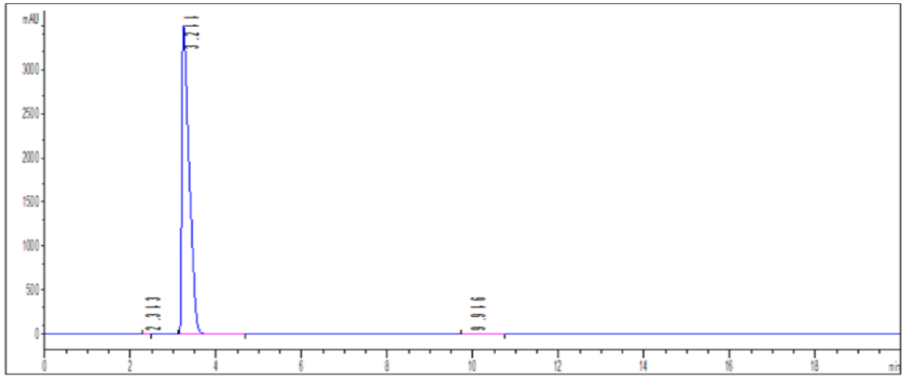

Retention time	Peak area	Peak height	Peak width	Symmetry factor	Relative peak area (%)
3.244	41540.5	3497.7	0.1735	0.294	99.906

B

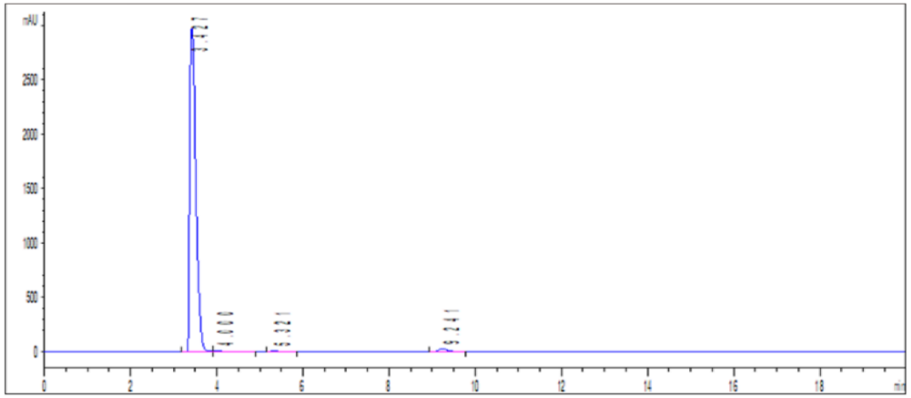

Retention time	Peak area	Peak height	Peak width	Symmetry factor	Relative peak area (%)
3.427	29400.6	2976.8	0.1531	0.535	98.155

Figure S2. HPLC chromatograms showing uric acid degradation by *Lactiplantibacillus plantarum* YC. (A) Chromatogram of the reaction mixture before incubation with strain YC. (B) Chromatogram after incubation with strain YC.

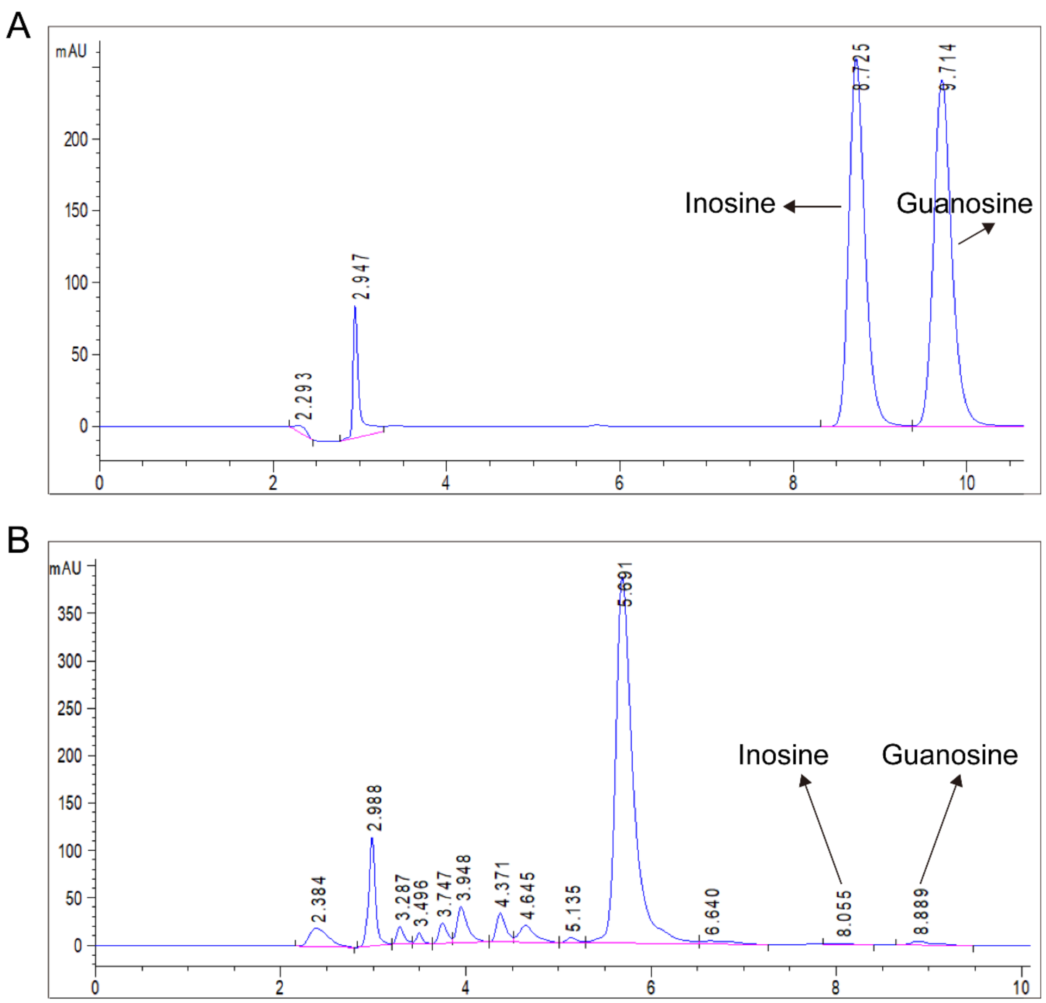

Figure S3. HPLC chromatograms showing the degradation of purine nucleosides by *Lactiplantibacillus plantarum* YC. (A) Chromatogram of the reaction mixture before incubation with strain YC. (B) Chromatogram after incubation with strain YC.
